# ATG5 Plays a Role in *Toxoplasma gondii* Replication and Egress Within Host Cells

**DOI:** 10.1155/tbed/4901030

**Published:** 2026-05-30

**Authors:** Fei Wu, Shanghua Wu, Qiying Ding, Zhiqiang Shi, Yinlong Liang, Hongchao Chen, Xiang Zhang, Jiawei Mi, Muxin Chen, Qianming Xu

**Affiliations:** ^1^ College of Veterinary Medicine, Anhui Agricultural University, Hefei, 230036, China, ahau.edu.cn; ^2^ College of Animal Sciences, Zhejiang Provincial Key Laboratory of Preventive Veterinary Medicine, Institute of Preventive Veterinary Medicine, Zhejiang University, Hangzhou, 310058, China, zju.edu.cn; ^3^ National Institute of Parasitic Diseases, Chinese Center for Disease Control and Prevention, Shanghai, 200025, China, chinacdc.cn

**Keywords:** ATG5, host–parasite interaction, mitochondrial dysfunction, premature, *Toxoplasma gondii*

## Abstract

*Toxoplasma gondii*, an intracellular parasite with a global distribution, causes toxoplasmosis, resulting in significant economic losses. Drug resistance and vaccine deficiency have spurred interest in identifying novel intervention targets, including autophagy and autophagy‐related proteins (ATGs). ATG5 is an important member of ATGs that plays a crucial role in disrupting the *T. gondii* parasitophorous vacuole membrane (PVM). However, its effects on the parasite’s intracellular lifecycle warrant further investigation. In this study, the transgenic Vero cells were constructed using a lentivirus‐mediated ATG5 overexpression or knockdown, which was used to evaluate the effects of ATG5 on *T. gondii* infection in vitro. The proliferation and development of the parasites in host cells were observed, and the number of parasitophorous vacuole (PV), pseudocysts, and lysed cells during various hours postinfection was counted under an optical microscope after Wright–Giemsa staining. The relative mRNA level of PTEN‐induced putative kinase 1 (PINK1) in infected cells was detected using a real‐time quantitative PCR (RT‐qPCR). The mitochondrial membrane potential (MMP) of *T. gondii* tachyzoites was evaluated with a JC‐1 fluorescence probe. The results showed that overexpressing ATG5 in Vero cells accelerated the invasion and egress processes of *T. gondii* tachyzoites and enhanced their proliferation compared to negative controls (NCs). Additionally, the mRNA level of PINK1 was upregulated in ATG5‐overexpressing cells. Crucially, ATG5 overexpression induced MMP depolarization in the parasites, probably leading to their mitochondrial dysfunction. These findings were corroborated by ATG5‐knockdown experiments, which yielded contrasting results. Collectively, ATG5 may have mediated premature parasite egress and mitochondrial damage, demonstrating its antiparasitic activity and therapeutic potential in controlling toxoplasmosis.

## 1. Introduction

*Toxoplasma gondii* is an obligate intracellular protozoan that is globally distributed, inhabits almost all host tissues, and causes toxoplasmosis [1, 2]. Felines serve as the definitive hosts, while nearly all warm‐blooded organisms (including humans, pets, livestock, and experimental animals) act as intermediate hosts [3, 4]. Approximately one‐third of the world’s population has been infected with or is currently infected with *T. gondii*. While most infections are asymptomatic, symptomatic cases might manifest as cervical lymphadenopathy, fever, fatigue, headache, muscular pain, or ocular diseases [5–8]. Critically, toxoplasmosis is life‐threatening to the foetus and immunocompromised individuals, primarily presenting as severe encephalitis [2, 9]. In animals, evidence shows high seropositivity of *T. gondii* in wildlife and free‐ranging livestock, which could induce abortion and stillbirth in pregnant animals, resulting in substantial economic losses [1, 2]. The medical and veterinary significance of toxoplasmosis has driven decades of research into interventions against *T. gondii* infection. While considerable progress has been made, no effective commercial vaccines are available [10, 11]. Sulfonamides remain the principal treatment strategy despite the emergence of drug‐resistant parasites [12, 13]. Thus, identifying novel vaccine candidates or drug targets remains a major focus of current research.

Autophagy, a conserved mechanism of maintaining cellular homeostasis, has recently emerged as a promising therapeutic strategy against toxoplasmosis [14, 15]. This tightly regulated process is mediated by highly conserved autophagy‐related proteins (ATGs) among host animals. Accumulating evidence indicates that autophagy and key ATGs, such as ATG3, ATG7, and the ATG12‐ATG5‐ATG16L1 complex, actively contribute to intracellular *T. gondii* clearance via multiple pathways targeting the parasitophorous vacuole membrane (PVM) [15, 16]. These ATGs, particularly ATG5, are essential for recruiting interferon γ (IFN‐γ)/LPS‐inducible effectors onto the *T. gondii* PVM, including ubiquitin‐like conjugation systems, LC3‐II, IFN‐γ‐inducible p47 GTPase IIGP1 (*Irga6*), and lysosomes [17]. These molecules disrupt the PVM through an autophagy‐dependent and/or‐independent manner to eliminate intracellular parasites. However, whether host ATGs affect *T. gondii* proliferation and development remains largely unknown, particularly Atg5, the required molecule for IFN‐γ/LPS‐dependent *T. gondii* clearance.

To this end, we generated transgenic Vero cell lines with high or low ATG5 levels to explore their effects on *T. gondii* infection. As well as detect the mRNA level of PTEN‐induced putative kinase 1 (PINK1) in transgenic cells and the mitochondrial membrane potential (MMP) of *T. gondii* tachyzoites to evaluate the effect of ATG5 on the mitochondrial function of host cells and parasites.

## 2. Materials and Methods

### 2.1. Parasite Culture

The *T. gondii* RH strain was preserved by the College of Veterinary Medicine at Anhui Agricultural University. Parasite culture was performed as previously described [18, 19]. In brief, the frozen tachyzoites of *T. gondii* were thawed in a 37°C water bath before reviving in Vero cells which were maintained in DMEM medium (HyClone, Logan, UT, USA) supplemented with 10% foetal bovine serum (FBS, HyClone), 2 mM glutamine, and 1% penicillin–streptomycin. Mature tachyzoites were purified by triturating the culture mixture (containing cells and parasites) using a 1 mL injector five times, followed by filtering with a 3 μm filter and resuspending in DMEM for counting and further use.

### 2.2. Transgenic Cell Line Construction and Verification

A lentivirus system was employed to generate transgenic cells expressing high or low levels of ATG5 following a well‐established method [20–22]. Three short hairpin RNA (shRNA) targeting the ATG5 coding sequence (CDS) were designed using BLOCK‐iT RNAi Designer (https://rnaidesigner.thermofisher.com/rnaiexpress/) according to *Chlorocebus sabaeus* ATG5 (Accession Number XM_008007460.1) and then synthesized and cloned into pHHsi‐hU6‐GFP‐Puro vector by Beijing Genomics Institute (BGI, Shenzhen, China). The most efficient one was selected for subsequent experiments. The corresponding CDS was amplified using a pair of primers (Table S1) and inserted into the pCDH vector. The recombinant vectors were co‐transfected into HEK 293T cells with psPAX2 and pMD2.G plasmids to generate lentivirus carrying either shRNA or ATG5 CDS, respectively. The viruses were collected from the cell culture medium and purified through centrifugation, followed by filtration using a 0.45 μm filter. These purified viruses were subsequently used to infect Vero cells to generate transgenic cell lines stably expressing ATG5 (overexpression group) or shRNA targeting ATG5 (RNAi group). Vero cells treated with the culture medium from HEK 293T cells co‐transfected with the empty vector (alongside psPAX2 and pMD2.G) served as a negative control (NC). All cells were maintained in DMEM medium containing 10% FBS and incubated at 37°C with 5% CO_2_. Puromycin was used for resistance selection. Total RNA from the selected cells was isolated using a Total RNA Kit I (Omega, Norcross, GA, USA) and reverse‐transcribed into cDNA using Reverse Transcriptase M‐MLV (Takara Bio Inc., Dalian, China). The relative transcriptional level of *ATG5* in acquired transgenic cells was assessed by real‐time quantitative PCR (RT‐qPCR) using the QuantiNova SYBR Green RT‐PCR Kit (Qiagen, Hilden, Germany) on a CFX96 Instrument (Biorad Inc., USA). The 2^−ΔΔCt^ method was employed to determine relative mRNA levels of the target gene [23]. GAPDH served as an internal control.

### 2.3. *T. gondii* Tachyzoites Replication and Egress Observation

Infection of *T. gondii* in Vero cells and sampling were performed as described [24, 25]. Briefly, cells were seeded in six‐well plates on coverslips with 2 mL of culture medium (DMEM supplemented with 10% FBS) at a density of 10^5^ cells per well. After 12 h, the medium was replaced, and an equivalent number of *T. gondii* tachyzoites were added. About 10 min later, noninvaded tachyzoites were removed through washing with 1 mL of DMEM three times. Samples were prepared at 0, 4, 8, 12, 24, 36, 48, 60, 72, 96, and 120 h postinfection through retrieving coverslips and staining with a Wright‐Giemsa Stain Kit (Abcam Inc., Toronto, ON, Canada). The prepared samples were observed under a Panthera I optical microscope (Motic China Group Co., Ltd., Hong Kong, China). For each sample, 100 fields of view were examined. In uninfected controls, the total number of cells per field was designated as *α*. After infection, uninfected cells (*β*) and tachyzoite‐containing cells (*γ*) were counted per field; the number of broken cells (indicating egress) was calculated as *α*−*β*−*γ*. Additionally, the numbers of parasitophorous vacuoles (PVs, containing 1–2 tachyzoites) and pseudocysts (vacuoles containing several to dozens of tachyzoites) were quantified.

### 2.4. Detection of the Transcription Level of PINK1

Cells expressing different ATG5 levels were collected at 0, 4, 12, 24, 36, 48, 60, and 72 h after challenging with *T. gondii* tachyzoites for total RNA isolation and cDNA synthesis, as described above. The relative mRNA level of *PINK1* in collected cells was detected by RT‐qPCR on a CFX96 Instrument (Biorad). The relative mRNA of GAPDH was detected to serve as an internal control. The primer set is listed in Table S1.

### 2.5. MMP Test

Following the above protocol, 10^5^ ATG5‐overexpressing or knockdown cells were individually seeded into six‐well plates and cultured overnight before incubation with equivalent numbers of *T. gondii* tachyzoites. After challenging, tachyzoites were harvested for JC‐1‐based MMP quantification following the manufacturer’s guidelines (Beyotime, Shanghai, China) and as previously described [26, 27]. In brief, free tachyzoites that had egressed were collected by centrifugation. The remaining cells were resuspended and mixed by pipetting and then disrupted using a 1 mL syringe needle to release intracellular tachyzoites. The suspension was filtered through a 5 μm filter to remove cells and debris, and the released tachyzoites were collected by centrifugation. Subsequently, the collected tachyzoites were washed, adjusted to 10^6^ tachyzoites/mL, and incubated with an identical volume of JC‐1 staining solutions (V/V = 1:1) at 37°C for 20 min in the dark. Parasites were then pelleted by centrifugation (3000 × *g*, 3 min, 4°C), washed twice with 1 mL of JC‐1 buffer, and analyzed on a flow cytometer (FACSCalibur, Becton Dickinson, USA). Fluorescence was captured from channels 1 (JC‐1 monomers; green) and 2 (J‐aggregates; red) and quantified using Flowjo software v10 (Becton Dickinson). MMP depolarization was measured as the ratio of red‐to‐green fluorescence intensity. Tachyzoites harvested from the NC group, processed identically, served as the irrelevant control.

### 2.6. Statistical Analysis

All experiments were performed in triplicate and repeated three times independently. Data were collected from three biological replicates and presented as mean ± standard deviation (SD). One‐way ANOVA or Student’s *t* test was performed using GraphPad Prism 8 software (Version 8.0.1, San Diego, CA, USA) for statistical analysis. *p* < 0.05 was considered statistically significant.

## 3. Results

### 3.1. Successfully Generates Transgenic Cell Lines

**Figure 1:**
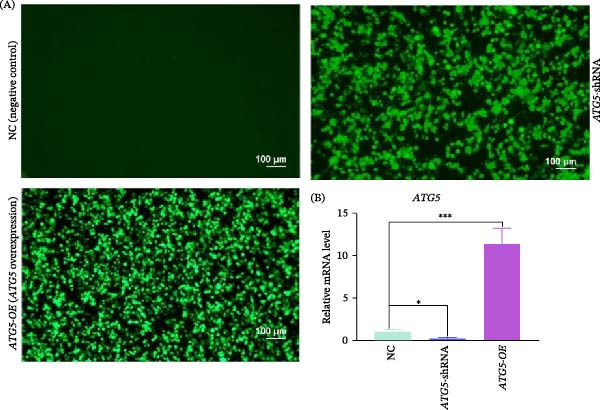
Generation of transgenic Vero cell lines using a Lentivirus‐mediated system. (A) lentiviruses production. HEK 293T cells were co‐transfected with psPAX2, pMD2.G and a recombinant plasmid containing either the ATG5 coding sequence (for overexpression) or *ATG5*‐targeting shRNA (for knockdown) to generate lentiviruses. Parental vector transfections served as negative control (NC). Green fluorescence intensity represents transfection efficiency, relating to viral production. The scale bar indicates 100 μm. (B) Relative *ATG5* mRNA level in Vero cells treated with different lentiviruses. Student’s *t* test was performed for statistical analysis. Error bars are shown as mean ± standard deviation (SD).  ^∗^ and  ^∗∗∗^ indicate *p* < 0.05 and *p* < 0.001, respectively.

The results showed that HEK 239T cells co‐transfected with packing, envelope, and recombinant transfer plasmids (containing *ATG5*‐shRNA or *ATG5*‐CDS) express abundant green fluorescent protein (Figure 1A), suggesting that considerable lentiviruses had been assembled successfully. Compared to the NC, the relative mRNA level of *ATG5* in transgenic Vero cells was decreased (*p* < 0.05) in the RNAi group and significantly increased (*p* < 0.001) in the overexpression group (Figure 1B).

### 3.2. ATG5 Accelerates the Invasion, Replication and Egress Processes of *T. gondii* in Vero Cells

**Figure 2:**
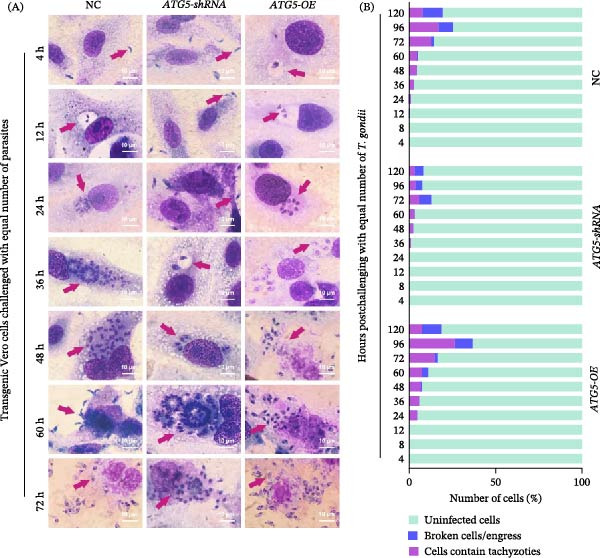
Effects of abnormal ATG5 expression in Vero cells on *Toxoplasma gondii* invasion, proliferation and egress. A, the hematoxylin–eosin staining of transgenic Vero cells at the indicated times post‐*T. gondii* infection. Red arrows indicate *T. gondii* tachyzoites. Scar bars are 10 μm. B, enumeration of transgenic Vero cells postinfection. Cells were categorized as uninfected cells, lysed, or *T. gondii*‐harboring. For each sample, 100 fields of view were examined. In uninfected controls, the total number of cells per field was designated as *α*. After infection, uninfected cells (*β*) and tachyzoite‐containing cells (*γ*) were counted per field; the number of broken cells (indicating egress) was calculated as *α*−*β*−*γ*.

After confirming the relatively low (RNAi group) and high (overexpression group) level of *ATG5* in transgenic Vero cells, these cell lines were subsequently used to evaluate the effects of *ATG5* dysregulation on *T. gondii* proliferation and development. The initial invasion of *T. gondii* tachyzoites, evidenced by PV formation (containing 1–2 *T. gondii* tachyzoites), was first observed at 4 h (overexpression group), 12 h (control group), and 36 h (RNAi group) postinfection (Figure 2A). Pseudocysts (PV containing several to dozens of tachyzoites) appeared at 24 h postinfection in both ATG5‐normal (NC) and ATG5‐overexpressing cells but were delayed until 48 h in ATG5‐knockdown cells. Furthermore, parasites in high‐ATG5 environments triggered host cell lysis (marked by tachyzoite release) earliest in overexpression cells (≤48 h) compared to wild‐type (60 h) and shRNA‐treated cells (72 h) (Figure 2A). Besides, cells containing parasites, uninfected cells, and lysed cells were counted; *T. gondii*‐infected cells were enumerated to assess parasite proliferation. Owing to their small size and transient existence, PVs formation was rarely observed (<0.5% of viable cells; Figure 2B), particularly following pseudocyst formation and host cell lysis. In groups with normal or high ATG5 levels, pseudocyst‐containing cells increased overtime, peaking at 96 h postinfection, while the proportion of lysed cells rose steadily. Conversely, the ATG5‐knockdown group exhibited significantly fewer both lysed cells and pseudocyst‐containing cells than other groups (Figure 2B). Obviously, ATG5 shortens the lifecycle of *T. gondii* tachyzoites in Vero cells, leading to their precocious differentiation.

### 3.3. ATG5 Maintains a Relatively Higher PINK1 Level in Vero Cells

**Figure 3:**
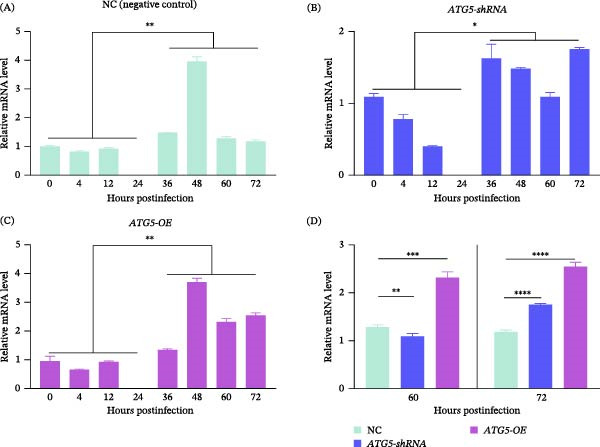
Relative *PINK1* mRNA level in transgenic Vero cells at 0 to 72 h after *T. gondii* infection. Cells from NC (A), *ATG5*‐shRNA (B), and ATG5 (C) group were evaluated. (D) The relative mRNA level of *PINK1* in infected cells when the parasites are egressing from host cells. Cells were detected after 60 and 72 h postinfection. One‐way ANOVA (A–C) or Student’s *t* test (D) was performed for statistical analysis. Error bars are mean ± SD.  ^∗^,  ^∗∗^,  ^∗∗∗^,  ^∗∗∗∗^ suggest *p* < 0.05, *p* < 0.01, *p* < 0.001, *p* < 0.0001, respectively.

Mitophagy is essential for maintaining mitochondrial homeostasis and thus participates in the host–pathogen interplay and the development of parasites in host cells [28, 29]. Therefore, we detected the expression of PTEN‐induced putative kinase 1 (PINK1), a key modulator of PINK1/parkin‐mediated mitophagy. PINK1 mRNA levels in *T. gondii*‐infected cells across all three groups (control, ATG5‐overexpressing, and ATG5‐knockdown) presented a consistent expression pattern: downregulation within 24 h and upregulation after 36 h (Figure 3A–C). Notably, PINK1 mRNA levels were significantly higher (*p* < 0.001) in ATG5‐overexpressing cells at 60 h and 72 h postinfection than those in control or knockdown cells (Figure 3D), presenting its possible role in *T. gondii* tachyzoites during egress.

### 3.4. ATG5 Promotes the MMP Depolarization of *T. Gondii*

**Figure 4:**
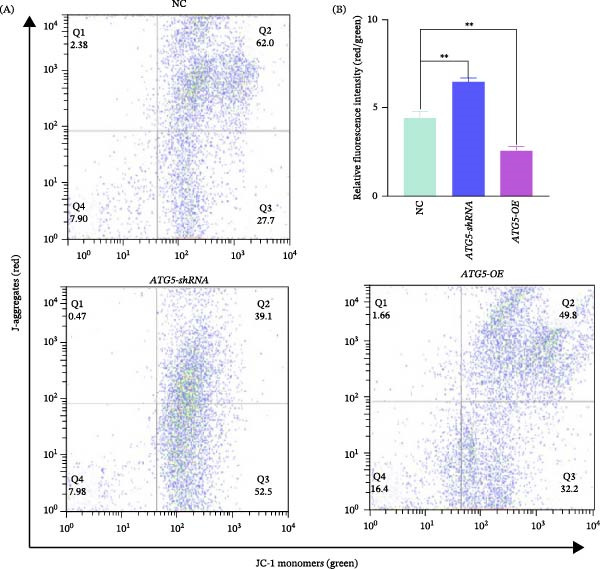
Mitochondrial membrane potential (MMP, Δψm) analysis of *T. gondii* tachyzoites using flow cytometry after JC‐1 probe staining. (A) The results of flow cytometry detection. Red fluorescence (JC‐1 aggregates) indicates normal Δψm, while green fluorescence (JC‐1 monomers) suggests depolarized Δψm. (B) Red‐to‐green fluorescence intensity. Student’s *t* test was performed for statistical analysis. Error bars are presented as mean ± SD.  ^∗∗^ imply *p* < 0.01.

To determine the impact of ATG5 on mitochondrial function, critical for *T. gondii* invasion [30], we quantified the MMP in tachyzoites. Compared to parasites cocultured with NC group cells, those exposed to ATG5‐overexpressing cells showed enhanced green fluorescence intensity (right‐shifted), while parasites cocultured with RNAi‐knockdown cells exhibited weakened green fluorescence (left‐shifted), particularly in quadrant 2 (Q2) of the scatter plot (Figure 4A). Correspondingly, relative to the NC, the MMP (red‐to‐green fluorescence ratio) in *T. gondii* significantly decreased in the overexpression group (*p* < 0.01) but increased obviously (*p* < 0.01) in the RNAi group (Figure 4B), indicating that ATG5 attenuated the freshly egressed tachyzoites.

## 4. Discussion

Toxoplasmosis is one of the most important neglected zoonotic diseases linked to poverty, which disturbs billions of people and animals worldwide and causes significant economic losses [1, 4, 5, 7, 9]. Moreover, the strategies against *T. gondii* infection are limited, including commercial vaccines and drugs, which provoke research on identifying intervention targets [10–13]. In this study, we generated transgenic Vero cell lines (expressing relatively high or low *ATG5* levels) to explore the effects of ATG5 on the lifecycle of *T. gondii* tachyzoites inside host cells.

Vero cells were used to generate transgenic cells for investigating ATG5 function. Normal people and animals are asymptomatic when infected with *T. gondii*, which is contributed by their well‐established native and acquired immune systems for killing most of the invading parasites [31–33]. For instance, macrophages and dendritic cells (DCs) modulate diverse biology processes (e.g., antigen presenting, cell survival, and cytokines secretion) through pattern recognition receptors (PRRs)‐mediated recognition and responses [34]. These cells also stimulate acquired immune responses mediated by T and B cell, which suppress parasite proliferation, promote clearance, and inhibit its transformation into bradyzoites [35]. Consequently, research on host mechanisms against *T. gondii* remains largely focused on immune cells, even though the parasite can infect nearly all nucleated cell types [17, 34, 35]. Given that tachyzoites may exhibit higher activity in nonimmune cells than in immune cells, the transgenic Vero cells generated in this study were used as a model to investigate the underlying mechanisms of host cell responses to *T. gondii* infection. Absolutely, further investigation should validate the findings from Vero cells in animal models.

ATG5 is proposed as a candidate target for intervention against *T. gondii*. Accumulated evidence indicates that the survival of intracellular parasites relies on active molecules derived from their host [36–38], including metabolites (i.e., squalene) and key genes (i.e., autophagy‐related genes). Furthermore, individual host gene modification represents a useful strategy for identifying key intervention targets and developing treatment approaches [37]. In the context of toxoplasmosis control, autophagy and associated proteins, including ATG3, ATG5, ATG7, and ATG8, are considered promising intervention targets [14, 15]. Among these, ATG5 is one of the key components of autophagy and performs diverse functions (e.g., antimicrobial activity) through both autophagosome‐dependent and independent manners [17, 39]. Although we found that ATG5 promotes MMP depolarization in *T. gondii* tachyzoites, a limitation of this study is that transmission electron microscopy was not performed, which would have provided more comprehensive results, particularly regarding morphological changes of organelles. Further investigation should focus on the damage caused by ATG5 to parasite mitochondria and their underlying mechanism.

ATG5 might induce premature and weakened vitality of *T. gondii* tachyzoites in Vero cells. Here, we observed that ATG5 overexpression promotes earlier invasion, enhances proliferation, and accelerates the release of *T. gondii* in Vero cells, thereby shortening the parasite’s intracellular residence time. In addition, the relative expression level of PINK1 was significantly (*p* < 0.05 or *p* < 0.01) higher at 60 and 72 h postinfection compared to the first 48 h. And PINK1 expression was also elevated in ATG5‐overexpressing cells relative to other groups beyond 48 h postinfection. Considering that PINK1 is a mitochondrial damage sensor in the PINK1‐Parkin system for quality control of mitophagy [40], ATG5 could upregulate the expression of PINK1 in host cells, suggesting that it may be involved in the mitophagy of *T. gondii* tachyzoites. However, whether and how ATG5 interacts with PINK1 and participates in parasite mitophagy remain unclear. These findings suggest that PINK1 might be associated with facilitating *T. gondii* egress, as tachyzoites predominantly invade and proliferate within the initial 48 h postinfection before typically egressing from host cells thereafter [41–44]. This is further supported by previous studies that host cell autophagy contributes to the development of apicomplexan protozoa [45, 46]. Moreover, enhanced mitochondrial depolarization was detected in tachyzoites maintained in ATG5‐overexpressing cells, implying that parasite mitochondrial dysfunction might impair subsequent invasion cycles [47]. We speculate that rapid egress of *T. gondii* tachyzoites results in earlier exposure of these parasites to host defences; the stronger depolarization of the membrane potential observed in earlier egressed tachyzoites points to mitochondrial damage, which would weaken their ability to reinvade. Therefore, even though parasite replication and egress are accelerated, we consider that ATG5 exerts an antiparasitic activity. Of course, these speculations require much more experimental evidence. Collectively, these results underscore the essential role of ATG5 in the infection and propagation of *T. gondii* within host cells, supporting its potential as a therapeutic target.

However, to fully verify that ATG5 rapidly exposes intracellular pathogens, prevents reinvasion, and thereby exerts antiparasitic activity, several key questions warrant further investigation, including: (1) Does host ATG5‐induced mitophagy facilitate the egress of *T. gondii* tachyzoites and inhibit subsequent invasion? (2) What is the precise mechanism by which ATG5 shortens the *T. gondii* lifecycle? (3) How does ATG5 destroy the MMP balance of *T. gondii*? (4) Does ATG5 impair long‐term *T. gondii* survival in vivo? Addressing these questions will provide critical insights into strategies for controlling chronic *T. gondii* infection and combating intracellular parasites.

## 5. Conclusions

In this work, we found that ATG5 could accelerate the invasion and replication of *T. gondii* tachyzoites, upregulate the PINK1 expression in host cells, and promote parasite mitochondrial depolarization, which paves the way to develop novel intervention strategies against *T. gondii* and other apicomplexan protozoa.

## Author Contributions

**Fei Wu**: conceptualization, methodology, investigation, formal analysis, writing – original draft. **Shanghua Wu**: methodology, investigation, writing – original draft. **Qiying Ding**: investigation, formal analysis, visualization. **Zhiqiang Shi**: investigation, data curation. **Yinglong Liang and Jiawei Mi**: investigation, visualization. **Hongchao Chen**: methodology, investigation. **Xiang Zhang**: investigation, validation. **Muxin Chen**: writing – review and editing. **Qianming Xu**: conceptualization, methodology, writing – review and editing.

## Funding

This work was supported by the High‐level Talent Introduction Program (Grant rc752501) of Anhui Agricultural University.

## Conflicts of Interest

The authors declare no conflicts of interest.

## Supporting Information

Additional supporting information can be found online in the Supporting Information section.

## Supporting information


**Supporting Information** Table S1: Primer sets used in this study.

## Data Availability

All data generated or analyzed during this study are fully contained within the manuscript and its supporting information files.
